# Health-Related Quality of Life in Patients With Advanced Endometrial Cancer Treated With Lenvatinib Plus Pembrolizumab or Treatment of Physician’s Choice

**DOI:** 10.1016/j.ejca.2023.03.015

**Published:** 2023-03-23

**Authors:** Domenica Lorusso, Nicoletta Colombo, Antonio Casado Herraez, Alessandro D. Santin, Emeline Colomba, David Scott Miller, Keiichi Fujiwara, Sandro Pignata, Sally E. Baron-Hay, Isabelle Laure Ray-Coquard, Ronnie Shapira-Frommer, Yong Man Kim, Mary McCormack, Rachid Massaad, Allison Martin Nguyen, Qi Zhao, Jodi McKenzie, Vimalanand S. Prabhu, Vicky Makker

**Affiliations:** a Fondazione Policlinico Universitario Agostino Gemelli IRCCS, and Catholic University of Sacred Heart, Rome, Italy; b University of Milan-Bicocca, European Institute of Oncology IRCCS, Milan, Italy; c Clínico San Carlos University Teaching Hospital, Madrid, Spain; d Yale University School of Medicine, New Haven, CT, USA; e Gustave Roussy Cancerology Institute, Villejuif, GINECO Group, France; f University of Texas Southwestern Medical Center, Dallas, TX, USA; g Saitama Medical University International Medical Center, Hidaka, Japan; h Istituto Nazionale Tumori IRCCS-Fondazione G. Pascale, Naples, Italy; i Royal North Shore Hospital, St Leonards, Australia; j Centre Léon Bérard, University Claude Bernard, Lyon, GINECO Group, France; k Sheba Medical Center, Ramat, Israel; l Asan Medical Center, University of Ulsan, Seoul, Korea, Republic of South Korea; m University College London Hospitals NHS Foundation Trust, London, United Kingdom; n MSD (Europe), Brussels, Belgium; o Merck & Co., Inc., Rahway, NJ, USA; p Eisai Inc., Nutley, NJ, USA; q Weill Cornell Medical Center, New York, NY, USA; r Memorial Sloan Kettering Cancer Center, New York, NY, USA

**Keywords:** Lenvatinib, Pembrolizumab, Patient-reported outcomes, Health-related quality of life, Endometrial cancer

## Abstract

**Purpose:**

Lenvatinib and pembrolizumab (LEN+PEMBRO) demonstrated clinically meaningful and statistically significant improvements in efficacy versus treatment of physician’s choice (TPC) in patients with advanced endometrial cancer (aEC) in the phase 3 Study 309/KEYNOTE-775. Health-related quality-of-life (HRQoL) is reported.

**Patients and Methods:**

Patients were randomly assigned to receive LEN+PEMBRO (n = 411; LEN 20 mg/day; PEMBRO 200 mg Q3W) or TPC (n = 416; doxorubicin 60 mg/m^2^ Q3W or paclitaxel 80 mg/m^2^ [weekly, 3 weeks on/1 week off]). Impact of treatment on HRQoL assessed by the global health status/quality of life (GHS/QoL) score of the European Organisation for Research and Treatment of Cancer Quality-of-Life Questionnaire (EORTC QLQ-C30) was a secondary objective; other scales of the Quality-of-Life Questionnaire (QLQ-C30), EORTC QLQ-Endometrial, 24 questions (EORTC QLQ-EN24), and EuroQoL 5 dimensions, 5 levels (EQ-5D-5L) were exploratory objectives. HRQoL was assessed on day 1 of each cycle. Completion/compliance, change from baseline, time to first and definitive deterioration were assessed. No multiplicity adjustments were applied for HRQoL endpoints.

**Results:**

The latest timepoint at which the predefined rates of completion (≥60%) and compliance (≥80%) were met was week 12. HRQoL at week 12 between treatment groups was generally similar. Time to first deterioration symptom scales favoured LEN+PEMBRO for QLQ-C30 dyspnoea, and QLQ-EN24 for poor body image, tingling/numbness, and hair loss; and TPC was favoured for QLQ-C30 pain, appetite loss, and diarrhoea, and QLQ-EN24 muscular pain. While the QLQ-C30 physical functional scale favoured TPC, other functional scales were generally similar between arms. Time to definitive deterioration favoured LEN +PEMBRO on most scales.

**Conclusion:**

HRQoL data from Study 309/KEYNOTE-775, with previously published efficacy and safety results, indicate that LEN+PEMBRO has an overall favourable benefit/risk profile versus TPC for the treatment of patients with aEC.

## 1. Introduction

Endometrial cancer (EC) is the fourth most common cancer in women and a common cause of cancer-related death both in the United States (US) and worldwide [1–3]. While most patients are diagnosed early, 10–15% of patients present with advanced disease, and the prognosis for these patients is poor because of the relatively few treatment options [2,3].

The efficacy and safety of lenvatinib (LEN) + pembrolizumab (PEMBRO) in patients with advanced EC were assessed in a phase 3 randomised study (Study 309/KEYNOTE-775) [4]. LEN +PEMBRO demonstrated statistically significant and clinically meaningful improvements versus treatment of physician’s choice (TPC; doxorubicin or paclitaxel) in progression-free survival (PFS, median; 7.2 months versus 3.8 months; hazard ratio [HR] 0.56, 95% confidence interval [CI], 0.47–0.66]) and overall survival (OS, median; 18.3 months versus 11.4 months, HR 0.62, 95% CI 0.51–0.75]) [4]. Safety data were generally consistent with the previously published safety data for the combination treatment in patients with advanced EC and across other tumour types [5–7].

Adverse events commonly associated with kinase inhibitors and/or immunotherapies include hypertension, immune reactions, rash, nausea, diarrhoea, fatigue, and musculoskeletal pain, and therefore treatment with these medications can have negative impacts on quality of life (QoL) [8–10]. With kinase inhibitors and immunotherapies becoming increasingly prevalent in the treatment of EC, the need for health-related QoL (HRQoL) data in patients with EC is crucial. Limited data are available on HRQoL in patients with EC, and the long-term impact on these patients has not been addressed [11].

Herein, we provide results from analyses of patient-reported outcomes (PROs) from Study 309/KEYNOTE-775 data using the European Organisation for Research and Treatment of Cancer (EORTC) Quality-of-Life Questionnaire (QLQ-C30), the EORTC QLQ-Endometrial, 24 questions (QLQ-EN24), and the EuroQoL 5 dimensions, 5 levels (EQ-5D-5L) instruments.

## 2. Methods

### 2.1. Study design and patients

Eligibility details have been included in the primary publication [4]. Briefly, patients had histologically confirmed advanced EC, with evidence of disease progression after one prior systemic platinum-based chemotherapy regimen in any setting for EC. Patients may have received up to one additional line of platinum-based chemotherapy if it was given in the neoadjuvant or adjuvant treatment setting. Prior hormonal therapies were not restricted.

Patients were randomly assigned 1:1 to receive LEN 20 mg once daily orally plus PEMBRO 200 mg intravenously once every 3 weeks (maximum of 35 administrations) or TPC (doxorubicin 60 mg/m^2^ once every 3 weeks [maximum lifetime cumulative dose of 500 mg/m^2^] or paclitaxel 80 mg/m^2^ given weekly [3 weeks on/1 week off]). Patients were first stratified by mismatch repair (MMR) status. Within the mismatch repair-proficient (pMMR) group, patients were further stratified by region, Eastern Cooperative Oncology Group performance status (ECOG PS), and prior history of pelvic radiation. HRQoL data were assessed for all patients who had received at least one dose of study treatment and completed at least one PRO assessment. Within this HRQoL full analysis set, HRQoL data were assessed for both the all-comer and pMMR groups.

Written informed consent was provided by all patients prior to undergoing any study-specific procedures. The study protocol was approved by relevant institutional review boards and was conducted in accordance with the principles of the World Medical Association Declaration of Helsinki.

### 2.2. PRO assessments

HRQoL was assessed by three instruments: the EORTC QLQ-C30, the EORTC QLQ-EN24, and the EQ-5D-5L. All these instruments have been well-validated [11–16]. Details of each instrument, and of the assessment schedule, are included in the Supplemental Material. HRQoL data were collected for completion and compliance, change from baseline, and time to deterioration. These are summarised in the Supplemental Material.

Data were collected for all scales on the three instruments throughout the study. However, based on clinical experience and United States’ Food and Drug Administration guidance [17], the authors have noted that the symptoms associated with the scales of QLQ-C30 fatigue, nausea/vomiting, appetite loss, and pain; and QLQ-EN24 urological, gastrointestinal, sexual/vaginal problems, and hair loss are among the most burdensome to patients with EC. For this reason, these scales were highlighted with Kaplan-Meier plots for time to first deterioration (TTfD) and time to definitive deterioration (TTdD). TTfD and TTdD analyses are further summarised in the Supplemental Material.

### 2.3. Study end-points

Primary end-points (OS and PFS by blinded independent central review per Response Evaluation Criteria In Solid Tumors v1.1) and safety have been previously published [4]. Evaluation of the impact of treatment on the global health status/quality of life (GHS/QoL) score of the EORTC QLQ-C30 was a secondary objective of this study. The impact of treatment on HRQoL by the EORTC QLQ-C30 (scales other than GHS/QoL), the EORTC QLQ-EN24, and the EQ-5D-5L were exploratory objectives. Within the HRQoL analyses, completion and compliance rates, changes in scores from baseline, TTfD, and TTdD were assessed.

### 2.4. Statistical analysis

Sample size and power calculations for Study 309/KEYNOTE-775 were estimated based on the primary end-points (OS and PFS) [4]. There was no formal hypothesis testing for the PRO analyses, thus power calculations were not performed; *P* values for all PRO analyses were 2-sided and nominal because there was no adjustment for multiplicity. PROs were analysed in the HRQoL full analysis set. The statistical analyses were performed using statistical procedures with SAS statistical software, version 9.4.

Completion and compliance of QLQ-C30, QLQ-EN24 and EQ-5D-5L by visit and by treatment were recorded.

The primary analysis timepoint for PRO analyses was prespecified (before database lock) in the statistical analysis plan as the latest timepoint at which PRO data for both groups were collected; the overall completion rate was at least 60%, and the overall compliance rate was at least 80%. Definitions for completion and compliance are included under Supplemental Material. To assess treatment effects on the change from baseline to the primary timepoint (week 12), a constrained longitudinal data analysis (cLDA) model [18] was applied, as described in the Supplemental Material.

Post hoc analyses were conducted for TTfD and TTdD and are described in the Supplemental Material.

## 3. Results

### 3.1. Patient baseline characteristics

**Fig. 1.**
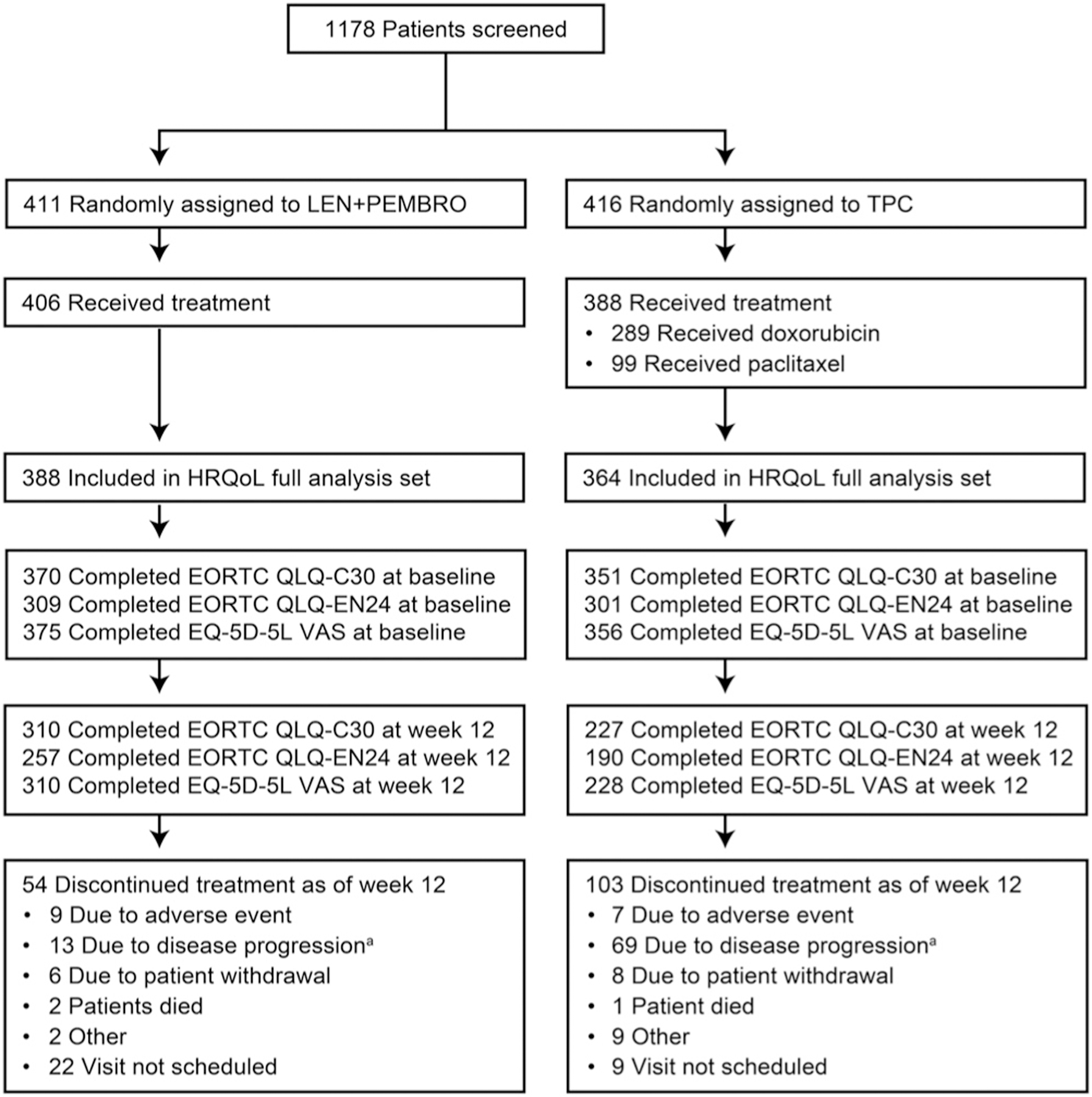
HRQoL Disposition. ^a^Includes clinical progression and progressive disease. European Organisation for Research and Treatment of Cancer; EN24, Endometrial, 24 questions; EQ-5D-5L, EuroQoL 5 dimensions, 5 levels; HRQoL, health-related quality of life; LEN, lenvatinib; PEMBRO, pembrolizumab; QLQ-C30, Quality-of-Life Questionnaire; TPC, treatment of physician’s choice.

Of patients included in Study 309/KEYNOTE-775, 411 were randomly assigned to LEN+PEMBRO and 416 were randomly assigned to TPC (Fig. 1).

Patient baseline characteristics have been previously reported [4] and patients were generally well-balanced between the treatment arms. Data herein will focus on the all-comer population, with pMMR data included in the Supplemental Material. At data cutoff (26th October 2020), among patients in the all-comer population, 69.5% in the LEN+PEMBRO arm and 73.5% in the TPC arm had discontinued treatment, with progressive disease as the most common reason for discontinuation.

### 3.2. Mean observation period

The mean observation period for patients in the LEN+PEMBRO arm was 9.3 months (standard deviation [SD], 6.2 months) for QLQ-C30 and EQ-5D-5L visual analog scale (VAS) questionnaires, and was 8.6 months (SD, 5.5 months) for QLQ-EN24. Contrastingly, patients in the TPC arm had a mean observation period of 4.3 months (SD, 2.9 months) for QLQ-C30 and EQ-5D-5L VAS questionnaires, and 4.2 months (SD, 2.8 months) for QLQ-EN24. Median duration of follow-up was 12.2 months (range 0.3–26.9) in the LEN+PEMBRO arm and 10.7 months (range 0.3–26.3) in the TPC arm.

### 3.3. Completion and compliance

All-comer population rates for completion and compliance of QLQ-C30, QLQ-EN24, and EQ-5D-5L VAS are shown in Table 1.

Week 12 was the latest timepoint at which the predetermined rates for completion (≥60%) and compliance (≥80%) were met (Table 1); therefore, this was defined as the primary timepoint for PRO analyses. Following this timepoint, completion and compliance rates decreased, particularly in the TPC arm.

### 3.4. Change from baseline

Baseline scores for the LEN+PEMBRO and TPC arms were similar (Table 2).

**Fig. 2.**
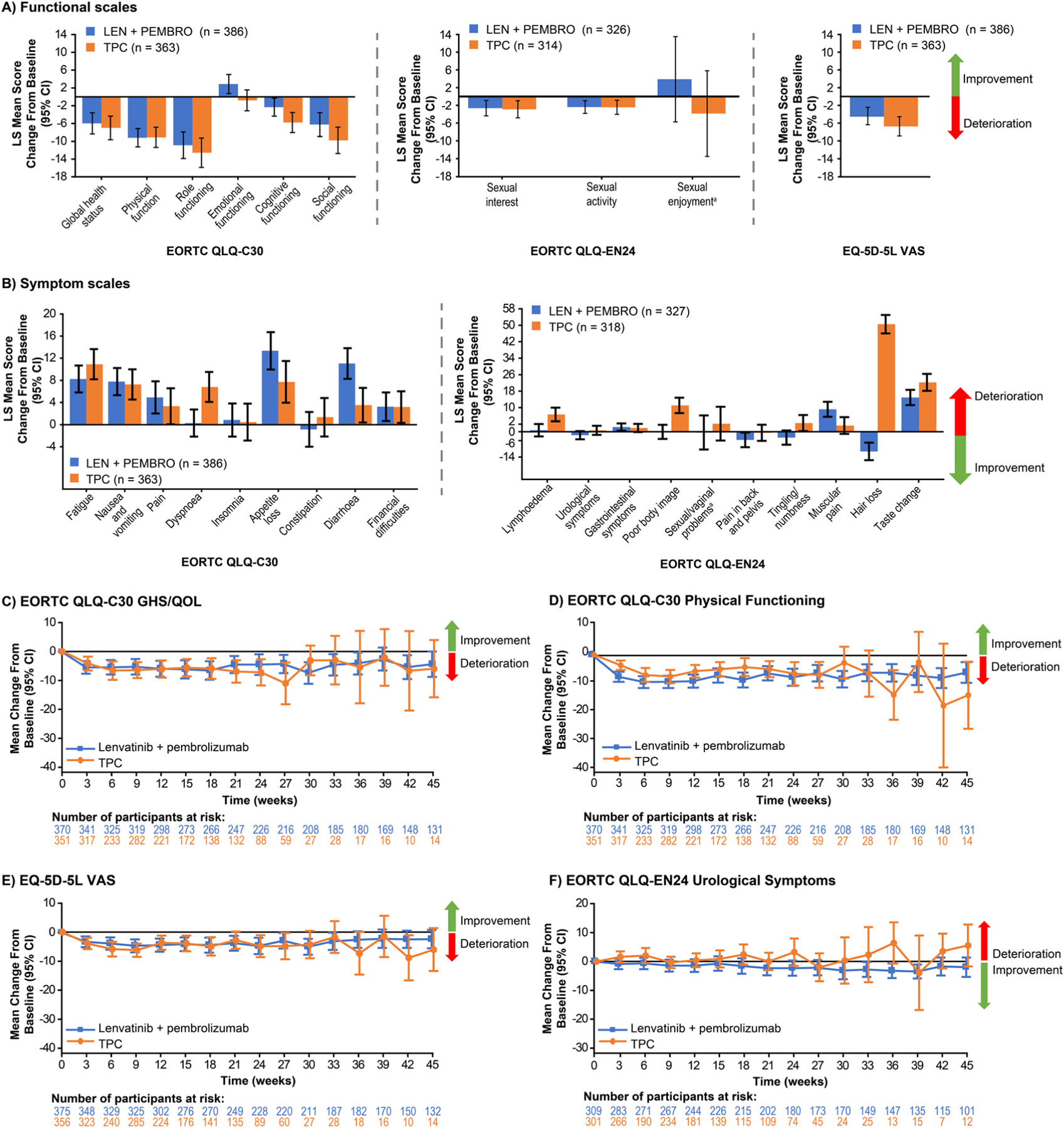
HRQoL Change From Baseline to Week 12 in the All-Comer Population at Week 12 (A, B) and Over Time (C-F). ^a^Patient numbers for the sexual enjoyment functional scale and the sexual/vaginal problems symptom scales are LEN+PEMBRO: n = 65, TPC: n = 55. CI, confidence interval; EORTC QLQ-C30, European Organisation for Research and Treatment of Cancer Quality-of-Life Questionnaire; EORTC QLQ-EN24, EORTC QLQ-Endometrial, 24 questions; EQ-5D-5L, EuroQoL 5 dimensions, 5 levels; GHS/QoL, global health status/quality of life; HRQoL, health-related quality of life; LEN, lenvatinib; LS, least squares; PEMBRO, pembrolizumab; TPC, treatment of physician’s choice; VAS, visual analog scale.

Changes from baseline to week 12 in the all-comer population are shown in Fig. 2A–B for the QLQ-C30 and QLQ-EN24 functional and symptom scales, and the EQ-5D-5L VAS. For the GHS/QoL scale, the least square mean changes from baseline to week 12 were −5.97 (95% CI −8.36, −3.58) in the LEN+PEMBRO arm and −6.98 (95% CI −9.63, −4.33) in the TPC arm (difference 1.01; 95% CI −2.28, 4.31; nominal *P* = 0.5460) (Table 2).

Most functional scales showed some deterioration from baseline to week 12, but declines were generally similar in both the LEN+PEMBRO and TPC arms. Scores on most of the QLQ-C30 and QLQ-EN24 symptom scales deteriorated in both groups with greater deterioration in the QLQ-C30 diarrhoea (non-overlapping CIs) and QLQ-EN24 muscular pain symptom scales among patients in the LEN+PEMBRO arm relative to TPC. A greater deterioration from baseline to week 12 among patients in the TPC arm relative to the LEN+PEMBRO arm was observed for the QLQ-C30 dyspnoea, QLQ-EN24 lymphoedema, QLQ-EN24 poor body image, and QLQ-EN24 hair loss scales (Fig. 2A–B). Changes from baseline to week 12 in the pMMR population for QLQ-C30 GHS/QoL, QLQ-C30 physical functioning, QLQ-EN24 urological symptoms, and EQ-5D-5L VAS are shown in Table S1 and Fig. S1.

When changes over time were assessed, no substantial changes were observed in either arm of the all-comer population for the QLQ-C30 GHS/QoL, QLQ-C30 physical functioning, EQ-5D-5L VAS, and QLQ-EN24 urological symptoms (Fig. 2C–F). Results for the pMMR population over time were generally similar to those of the all-comer population (Fig. S1).

### 3.5. Time to first deterioration

**Fig. 3.**
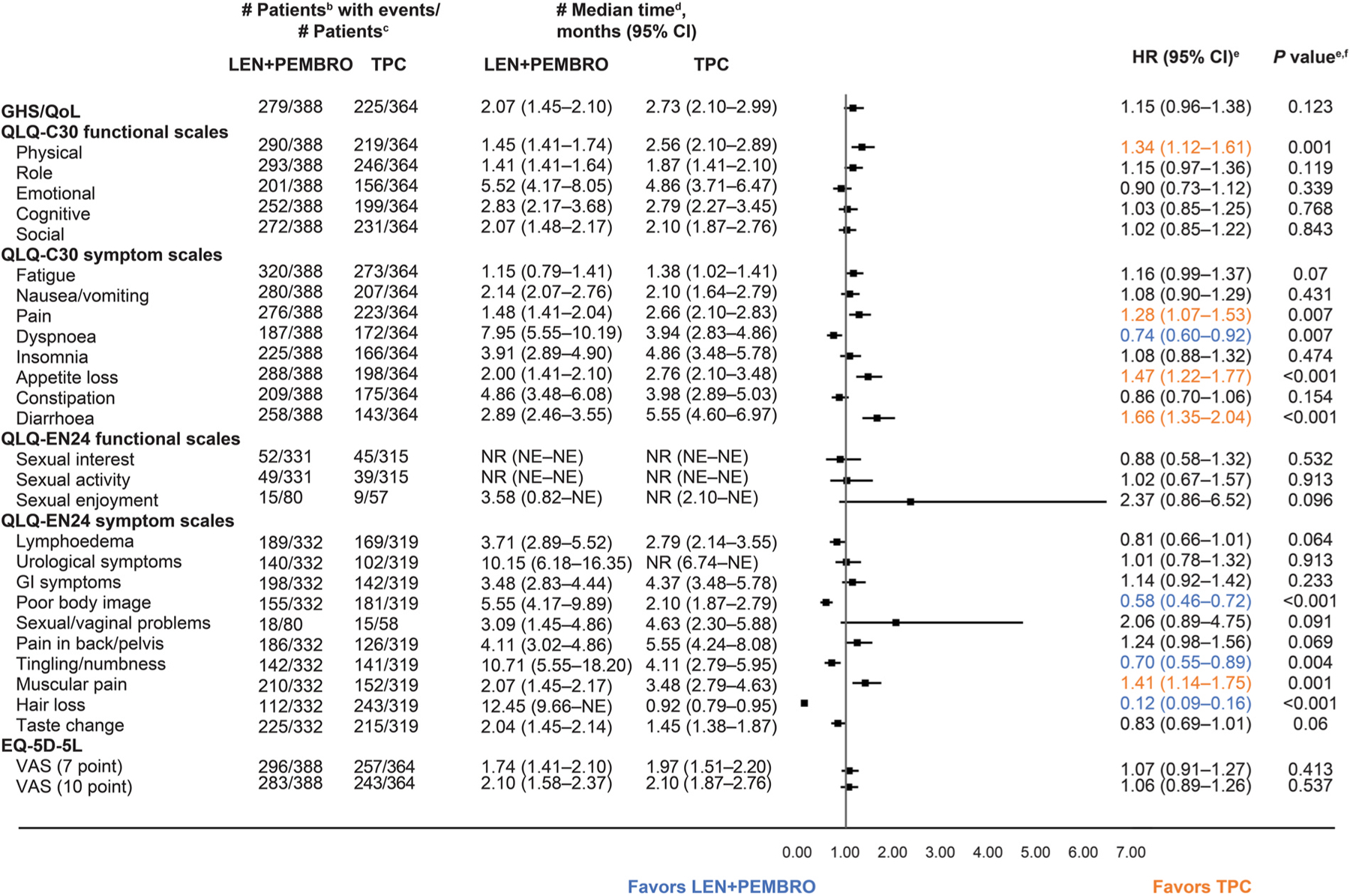
Time to First Deterioration in the All-Comer Population^a^. ^a^Database cutoff: 26th October 2020. ^b^Time to first deterioration is defined as the time from first dose of treatment to first onset of ≥10 points decrease from baseline for functional scales (decrease of ≥7 points for the for VAS 7-point threshold) and ≥10 points increase for symptom scales; a longer time to deterioration is considered more favourable. ^c^Number of patients in the HRQoL full analysis set with available data. ^d^From product-limit (Kaplan-Meier) method for censored data. ^e^Based on Cox regression model with treatment as a covariate stratified by MMR status, ECOG PS, geographic region, and prior history of pelvic radiation. ^f^Two-sided *P* value using Wald test (score test in case of zero event in 1 treatment group). CI, confidence interval; ECOG PS, Eastern Cooperative Oncology Group performance status; EORTC QLQ-C30, European Organisation for Research and Treatment of Cancer Quality-of-Life Questionnaire; EORTC QLQ-EN24, EORTC QLQ-Endometrial, 24 questions; EQ-5D-5L, EuroQoL 5 dimensions, 5 levels; GHS/QoL, global health status/quality of life; GI, gastrointestinal; LEN, lenvatinib; MMR, mismatch repair; NE, not estimable; NR, not reached; PEMBRO, pembrolizumab; TPC, treatment of physician’s choice; VAS, visual analog scale.

In the all-comer population, comparisons of TTfD on the functional scales between LEN+PEMBRO and TPC were generally similar; however, TTfD results for the QLQ-C30 physical functioning scale nominally significantly favoured TPC (Fig. 3).

**Fig. 4.**
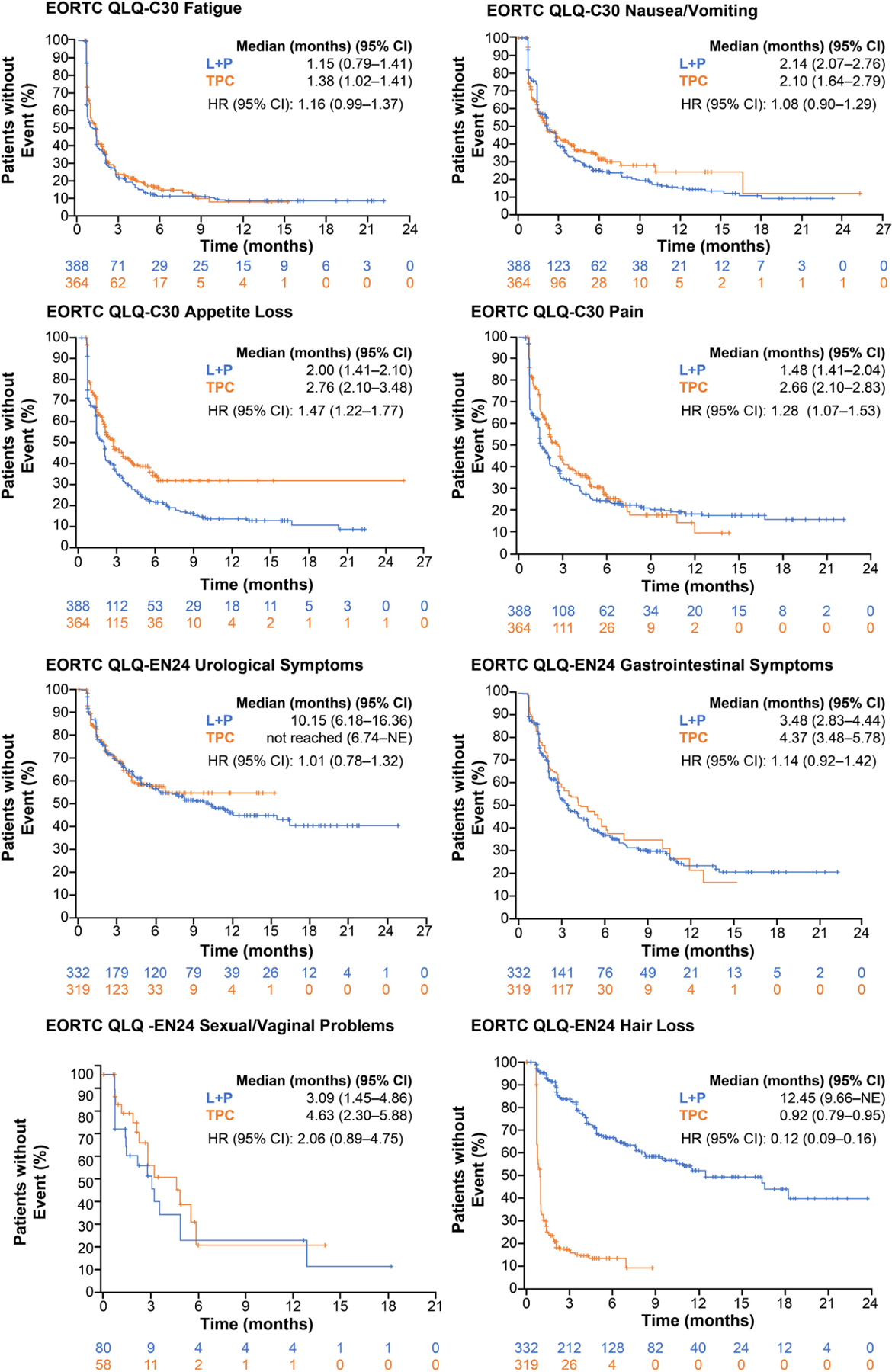
Time to First Deterioration for Selected Scales of Interest in the All-Comer Population. CI, confidence interval; EORTC QLQ-C30, European Organisation for Research and Treatment of Cancer Quality-of-Life Questionnaire; EORTC QLQ-EN24, EORTC QLQ-Endometrial, 24 questions; HR, hazard ratio; L, lenvatinib; P, pembrolizumab; NE, not estimable; TPC, treatment of physician’s choice.

Within the symptom scales, TTfD results nominally significantly favoured LEN+PEMBRO over TPC for QLQ-C30 dyspnoea, QLQ-EN24 poor body image, QLQ-EN24 tingling/numbness, and QLQ-EN24 hair loss. TTfD for QLQ-C30 pain, QLQ-C30 appetite loss, QLQ-C30 diarrhoea, and QLQ-EN24 muscular pain nominally significantly favoured TPC over LEN+PEMBRO (Fig. 3). Kaplan-Meier plots of TTfD in the all-comer population for the symptom scales of particular clinical interest are included in Fig. 4.

### 3.6. Time to definitive deterioration

**Fig. 5.**
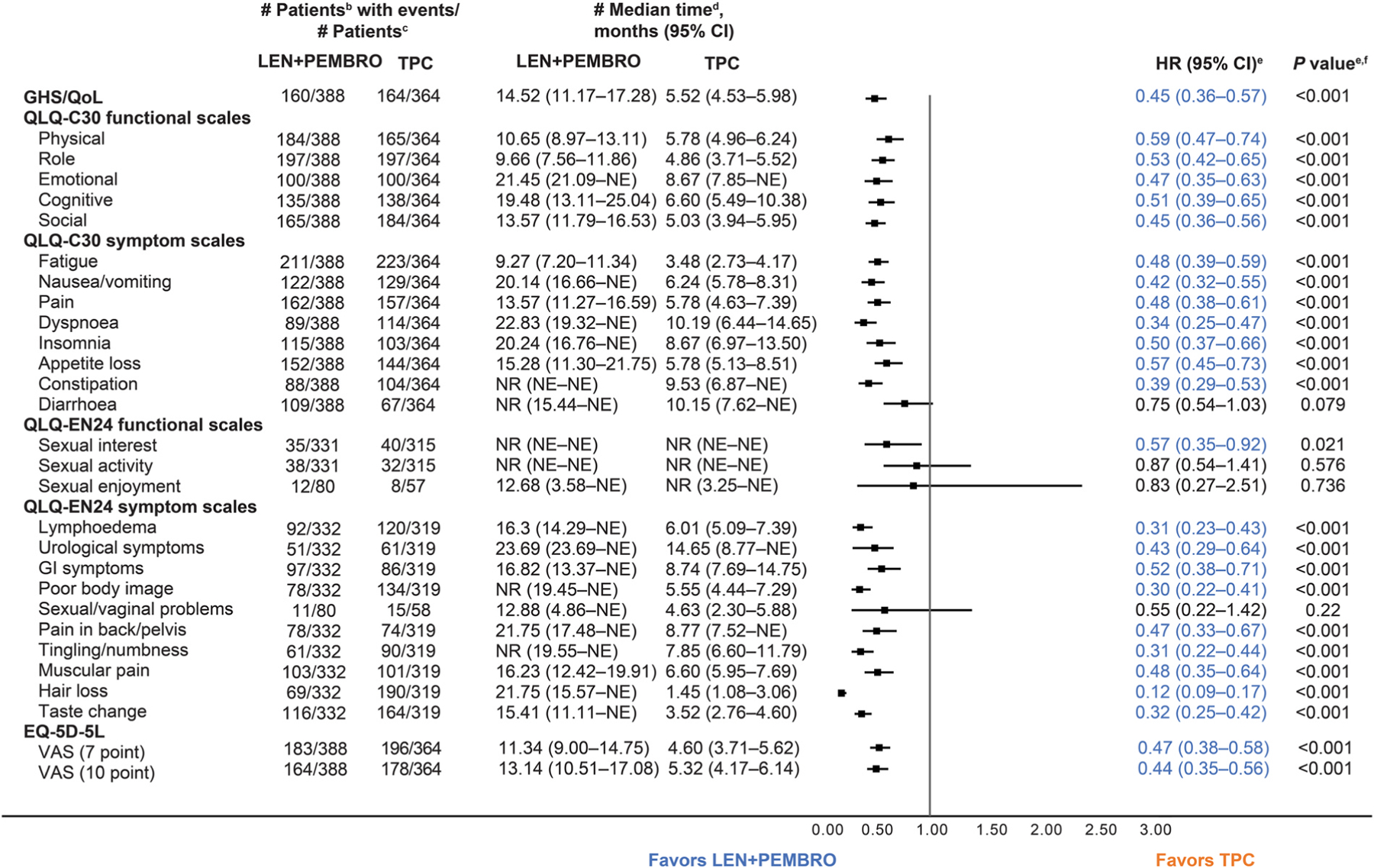
Time to Definitive Deterioration in the All-Comer Population^a^. ^a^Database cutoff: 26th October 2020. ^b^Time to definitive deterioration is defined as the time from first dose of treatment to first onset of ≥10 points decrease from baseline for functional scales (decrease of ≥7 points for the for VAS 7-point threshold) and ≥10 points increase for symptom scales from baseline without subsequent recovery or no subsequent assessment data; a longer time to deterioration is considered more favourable. ^c^Number of patients in the HRQoL full analysis set with available data. ^d^From product-limit (Kaplan-Meier) method for censored data. ^e^Based on Cox regression model with treatment as a covariate stratified by MMR status, ECOG PS, geographic region, and prior history of pelvic radiation. ^f^Twosided *P* value using Wald test (score test in case of zero event in 1 treatment group). CI, confidence interval; ECOG PS, Eastern Cooperative Oncology Group performance status; EORTC QLQ-C30, European Organisation for Research and Treatment of Cancer Quality-of-Life Questionnaire; EORTC QLQ-EN24, EORTC QLQ-Endometrial, 24 questions; EQ-5D-5L, EuroQoL 5 dimensions, 5 levels; GHS/QoL, global health status/quality of life; GI, gastrointestinal; LEN, lenvatinib; MMR, mismatch repair; NE, not estimable; NR, not reached; PEMBRO, pembrolizumab; TPC, treatment of physician’s choice; VAS, visual analog scale.

Among the all-comer population, median TTdD was numerically longer for patients in the LEN+PEMBRO arm relative to the TPC arm for most PRO scales (Fig. 5).

**Fig. 6.**
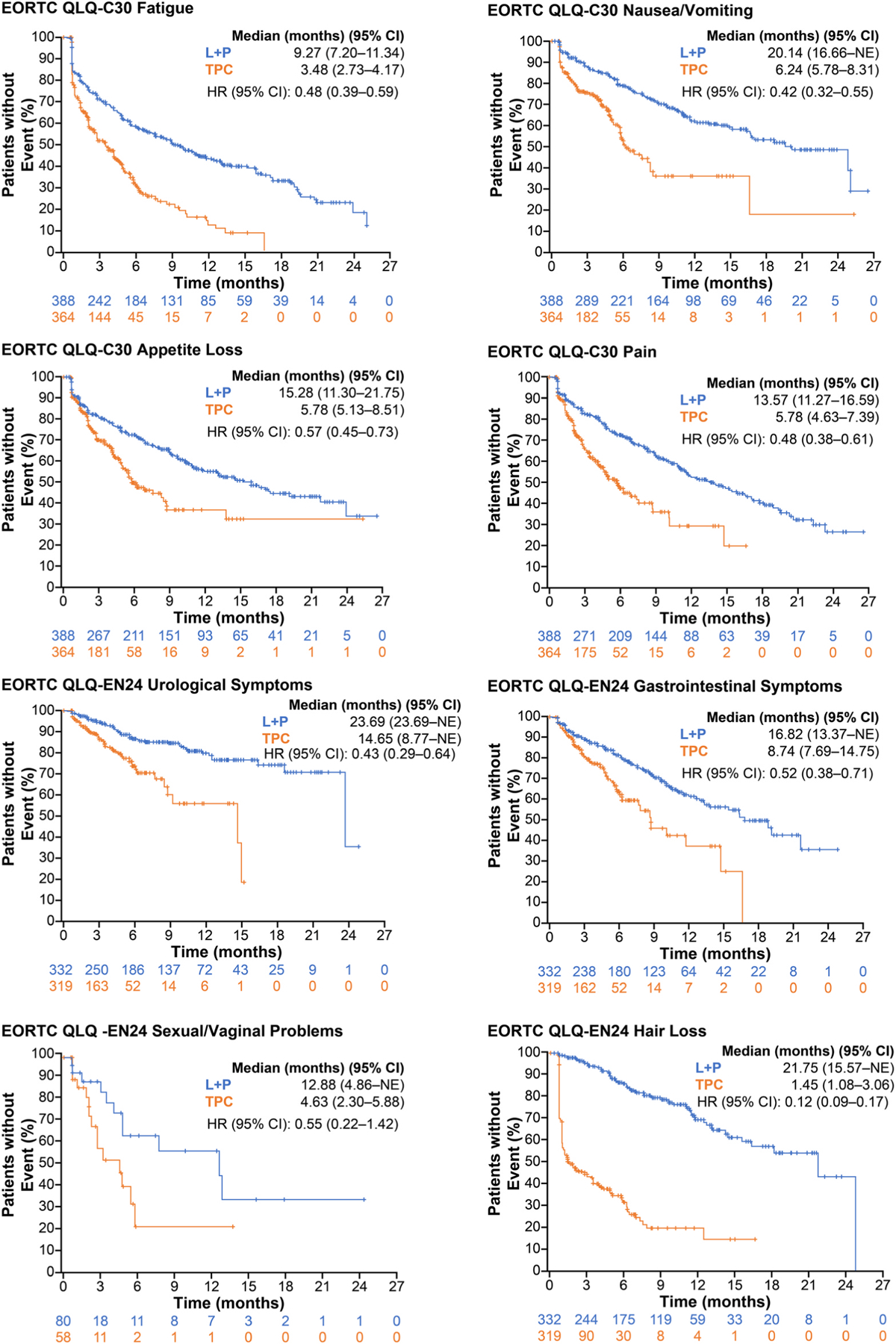
Time to Definitive Deterioration for Selected Scales of Interest in the All-Comer Population. CI, confidence interval; EORTC QLQ-C30, European Organisation for Research and Treatment of Cancer Quality-of-Life Questionnaire; EORTC QLQ-EN24, EORTC QLQ-Endometrial, 24 questions; HR, hazard ratio; L, lenvatinib; NE, not estimable; P, pembrolizumab; TPC, treatment of physician’s choice.

Longer TTdD in the LEN+PEMBRO arm was nominally significant for the QLQ-C30 GHS/QoL and the QLQ-C30 functional scales and most of the QLQ-C30 symptom scales, as well as the QLQ-EN24 sexual interest scale and most of the QLQ-EN24 symptom scales. In addition, TTdD for the EQ-5D-5L VAS also nominally significantly favoured LEN+PEMBRO versus TPC. No scales nominally significantly favoured TPC versus LEN+PEMBRO. Scales of particular clinical interest are shown in Kaplan-Meier plots in Fig. 6.

## 4. Discussion

In this analysis of PROs in patients with advanced EC treated with either LEN+PEMBRO or TPC, deterioration in HRQoL was observed over time, but declines were generally similar in both treatment groups (based on cLDA analysis). Differences could be seen on individual scales: appetite loss, diarrhoea, and muscular pain all appeared worse with LEN+PEMBRO compared with TPC; whereas dyspnoea, poor body image, and hair loss appeared worse with TPC compared with LEN+PEMBRO. Together, these differences in individual PRO outputs can help explain how scores on the more broad GHS/QoL instrument were generally similar between treatment arms, while patients’ HRQoL on individual symptom scales varied depending on the specific study treatment. Results from the pMMR population were generally consistent with those from the all-comer population. Longer longitudinal follow-up on QLQ-C30 GHS/QoL and physical functioning, as well as EQ-5D-5L scores, were also consistent across both arms.

Although overall TTfD data did not demonstrably favour either arm, when TTdD was assessed, LEN+PEMBRO was favoured versus TPC for almost all scales. TTfD (thought to be linked to treatment toxicity) typically favours the treatment with a shorter observation period (i.e. TPC) as potential first deterioration events can be missed if none are detected prior to early discontinuation. Alternatively, TTdD typically favours the treatment with a relatively longer observation period (i.e. LEN+PEMBRO) as patients in this group have a higher chance of temporary recovery, leading to lack of events for definitive deterioration. This is particularly relevant in this study where the mean observation period for LEN+PEMBRO was more than twice the mean observation period for TPC; however, a strength of our study design is that patients were asked to complete the HRQoL questionnaires during post-treatment follow up, for the equivalent of four cycle lengths. Also, the increased efficacy of LEN+PEMBRO versus TPC, with significantly higher tumour responses and PFS, likely contributes to increased time on treatment and, hence, improved recovery in PROs after initial deterioration.

This hypothesis is further supported by other phase 3 trials that assessed time to deterioration and also showed longer TTdD in treatment arms with significantly or numerically favourable efficacy. These results were consistent across indications including renal cell carcinoma (in a trial of LEN in combination with PEMBRO or everolimus compared with sunitinib) [7,19], hepatocellular carcinoma (in a non-inferiority trial comparing LEN vs sorafenib) [20,21], and a pooled analysis of four trials comparing regorafenib versus placebo across three tumour types [22].

In the context of efficacy and safety data [4], the PRO data derived from Study 309/KEYNOTE-775 strongly suggest that LEN+PEMBRO offers substantial benefits for the treatment of patients with advanced EC. Efficacy results favoured LEN+PEMBRO over TPC (median PFS hazard ratio [HR], 0.56 [95% CI 0.47–0.66]; *P* < 0.001; median OS HR, 0.62 [95% CI 0.51–0.75]; *P* < 0.001) [4]. Among patients in the safety analysis population in the LEN+PEMBRO arm, median duration of treatment was 231 days compared with 104.5 days in the TPC arm [4]. Treatment-emergent adverse events (TEAEs) observed in the LEN+PEMBRO arm of Study 309/KEYNOTE-775 were generally consistent with those observed in previous studies [4–6], and with LEN and PEMBRO monotherapies [23,24]. PROs for both arms over the first 12 weeks of treatment were generally similar overall, though differences were seen in a few specific scales, indicating that patient experience was generally similar. When viewed in context of the improved efficacy and longer duration of treatment, these PRO results support administration of LEN+PEMBRO over TPC in patients with EC.

One limitation of these analyses is that while the HRQoL secondary and exploratory end-points were predefined, no multiplicity adjustments were applied for these end-points and, therefore, *P*-values should be considered nominal and descriptive. Furthermore, the time to deterioration analyses were conducted post hoc and all related statistics should be considered nominal. Also, this was an open-label study, which could have potentially biased the results. These data were collected within the structure of a controlled clinical trial, with specific inclusion and exclusion criteria, which could limit the applicability to real-world populations. While clinical trial data are valuable to demonstrate the impact of treatments on PROs, data collected in the real-world setting would be helpful to supplement the HRQoL data observed in Study 309/KEYNOTE-775.

Another limitation is that there were differences in completion and compliance rates between the LEN +PEMBRO and TPC arms, which may have affected the observed results. This is particularly evident at later time points, as patient discontinuation limited the availability of data over time. For this reason, any long-term impact beyond treatment discontinuation is difficult to discern. Moreover, some types of therapy have lifetime dose limits (i.e. doxorubicin was limited to a cumulative dose of 500 mg/m^2^ and PEMBRO was limited to 200 mg every 3 weeks for up to 35 administrations, with potential eligibility for an additional 17 administrations), which should be considered in the context of long-term treatment. Of note, HRQoL was assessed on the first day of each cycle, but cycles were different lengths (21 days for LEN+PEMBRO and doxorubicin and 28 days for paclitaxel). Analyses for combination treatment regimens can be challenging, particularly for combinations in which one drug is administered daily and another once every 3 weeks. Relatedly, it can be difficult to compare daily therapy (i.e. LEN) with sequential therapy (i.e. doxorubicin or paclitaxel).

Studies have shown that both patients and clinicians find collection and discussion of PRO data to be valuable components of cancer therapy [25,26]. Given that patients with EC often face physical challenges such as advanced age, obesity, and pre-existing comorbidities (including diabetes or hypertension) that can impact their cancer treatment and their QoL [2,3,11], HRQoL studies in this patient population are critical. Unfortunately, data on HRQoL outcomes in patients with EC are limited, and patients with advanced EC are particularly under-represented in the literature [11]. In a phase 3 trial in patients with advanced EC, HRQoL results favoured carboplatin plus paclitaxel over paclitaxel-doxorubicin-cisplatin [27], but there are few other studies in patients with advanced EC with HRQoL data. Therefore, this analysis of patients with advanced EC treated with LEN+PEMBRO versus TPC is particularly important and, to our knowledge, represents the first HRQoL data in this patient population and setting.

Data from this analysis of PROs from Study 309/KEYNOTE-775 demonstrated that from baseline to week 12, HRQoL (as seen in the QLQ-C30 GHS/QoL, functional and symptom scales of the QLQ-C30 and the QLQ-EN24, and the EQ-5D-5L) showed deterioration in patients with EC treated with LEN+PEMBRO. While deteriorations on specific scales were different between arms, declines in HRQoL also occurred in the TPC arm, resulting in only minor differences between LEN+PEMBRO and TPC overall. Given the clinically meaningful and statistically significant improvement in PFS, OS, and objective response rate, and a safety profile that was consistent with previously reported studies [4–6], these PRO data further indicate that LEN+PEMBRO has an overall favourable benefit/risk profile compared with TPC for the treatment of patients with advanced EC. We conclude that LEN+PEMBRO represents a new standard of care for patients with advanced or recurrent EC following prior systemic therapy in any setting, and should be considered as a first option for patients with advanced EC.

## Supplementary Material

1

## Figures and Tables

**Table 1:** HRQoL Instrument Completion and Compliance Rates in the All-Comer Population.

Treatment visit	Category, rate %	EORTC QLQ-C30		EORTC QLQ-EN24^a^		EQ-5D-5L VAS	
		L+P	TPC	L+P	TPC	L+P	TPC
		n = 388	n = 364	n = 388	n = 364	n = 388	n = 364
**Baseline**	Completion	95.4	96.4	79.6	82.7	96.6	97.8
	Compliance	95.9	96.7	80.1	82.9	97.2	98.1
**Week 12**	Completion	79.9	62.4	66.2	52.2	79.9	62.6
	Compliance	92.8	87.0	77.6	72.8	92.8	87.4
**Week 24**	Completion	59.8	24.5	49.0	20.9	59.8	24.7
	Compliance	88.9	73.6	72.8	63.3	88.9	74.4
**Week 36**	Completion	47.2	4.9	39.2	3.8	47.4	4.9
	Compliance	88.4	72.0	73.8	56.0	88.9	72.0

The completion rate at a specific timepoint was defined as the number of patients who completed at least one item at that specific timepoint divided by the number of patients in the HRQoL full analysis set. The compliance rate was defined as the number of patients who completed at least one item at the specific timepoint divided by the number of patients expected to complete the PRO assessment at that visit, not including patients missing by design (i.e. death, discontinuation, translation not available). Median duration of treatment (among patients in the safety analysis set; L+P: n = 406, TPC: n = 388) was 231 days in the L+P arm and 104.5 days in the TPC arm. EORTC QLQ-C30, European Organisation for Research and Treatment of Cancer Quality-of-Life Questionnaire; EORTC QLQ-EN24, EORTC QLQ-Endometrial, 24 questions; EQ-5D-5L, EuroQoL 5 dimensions, 5 levels; HRQoL, health-related quality of life; L+P, lenvatinib + pembrolizumab; PRO, patient-reported outcome; TPC, treatment of physician’s choice; VAS, visual analog scale.

a Translation of the EORTC QLQ-EN24 questionnaire was not available in some sites at the start of study enrolment, resulting in lower completion rates compared with the other questionnaires.

**Table 2:** HRQoL Change From Baseline to Week 12 in the All-Comer Population.

Parameter	LEN+PEMBRO			TPC			Difference in LS means (95% CI)^a^	*P*-value
	Baseline mean (SD)	Week 12 mean (SD)	Change from baseline to week 12 LS mean (95% CI)	Baseline mean (SD)	Week 12 mean (SD)	Change from baseline to week 12 LS mean (95% CI)		
**EORTC QLQ-C30 GHS/QoL**	65.74 (21.87)	60.56 (21.35)	−5.97 (−8.36, −3.58)	65.69 (22.71)	62.70 (21.08)	−6.98 (−9.63, −4.33)	1.01 (−2.28, 4.31)	0.5460
**EORTC QLQ-C30 Physical Functioning**	78.68 (20.08)	71.51 (21.12)	−9.19 (−11.24, −7.14)	75.97 (20.88)	71.92 (21.78)	−9.10 (−11.37, −6.83)	−0.09 (−3.08, 2.90)	0.9537
**EORTC QLQ-EN24 Urological Symptoms**	14.89 (17.94)	12.91 (18.76)	−1.62 (−3.56, 0.31)	16.00 (19.32)	16.18 (18.33)	0.66 (−1.47, 2.79)	−2.29 (−5.03, 0.45)	0.1014
**EQ-5D-5L VAS**	73.70 (18.24)	70.37 (18.31)	−4.44 (−6.43, −2.46)	73.53 (18.91)	70.61 (19.25)	−6.79 (−8.98, −4.60)	2.35 (−0.44, 5.14)	0.0991

CI, confidence interval; EORTC QLQ-C30, European Organisation for Research and Treatment of Cancer Quality-of-Life Questionnaire; EORTC QLQ-EN24, EORTC QLQ-Endometrial, 24 questions; EQ-5D-5L, EuroQoL 5 dimensions, 5 levels; GHS/QoL, global health status/quality of life; HRQoL, health-related quality of life; LEN+PEMBRO, lenvatinib+pembrolizumab; LS, least squares; SD, standard deviation; TPC, treatment of physician’s choice; VAS, visual analog scale.

a Based on a constrained longitudinal data analysis model with the patient-reported outcome scores as the response variable with covariates for treatment by study visit interaction, stratification factors mismatch repair status, Eastern Cooperative Oncology Group performance status, geographic region, and prior history of pelvic radiation.
