# Supplementary material for: Health-Related Quality of Life in Patients With Advanced Endometrial Cancer Treated With Lenvatinib Plus Pembrolizumab or Treatment of Physician’s Choice
Source: Eur J Cancer. Author manuscript; Available in PMC 2024 Apr 9. (PMC11003310; doi:10.1016/j.ejca.2023.03.015)
Supplement: 1 [file NIHMS1936689-supplement-1.docx]

**Supplemental Material**

The supplemental material consists of methodological details that enhance understanding of the instruments used in this analysis and the procedures implemented. It also includes change from baseline data for the pMMR patient group.

*PRO instruments*

| EORTC QLQ-C30 Instrument | EORTC QLQ-EN24 | EQ-5D-5L Instrument |
| --- | --- | --- |
| Purpose: To measure the core set of patient-reported outcomes recommended by the FDA, including physical, role, and disease-related symptoms | Purpose: Designed to assess disease- and treatment-specific aspects of HRQoL in patients with all stages of EC and at different phases of treatment | Purpose: To measure health status across multiple disease types and to compute utility weights for cost-effectiveness analyses in Europe and other countries |
| Includes a global health status, functional and symptom scales | Includes functional and symptom scales | Visual analog scale (VAS) |
| - Global Health Status/Quality of Life scale - Functional scales   1. Physical   2. Role   3. Emotional   4. Cognitive   5. Social - Symptom scales   1. Fatigue   2. Nausea and vomiting   3. Pain   4. Dyspnea   5. Insomnia   6. Appetite loss   7. Constipation   8. Diarrhea   9. Financial difficulties | - Functional scales   1. Sexual interest   2. Sexual activity   3. Sexual enjoyment - Symptom scales   1. Lymphoedema   2. Urological symptoms   3. Gastrointestinal symptoms   4. Poor body image   5. Sexual/vaginal problems   6. Pain in back and pelvis   7. Tingling/numbness   8. Muscular pain   9. Hair loss   10. Taste change | - 5 Dimensions   1. Mobility   2. Self-care   3. Usual activities   4. Pain/discomfort   5. Anxiety/depression - 5 Levels   1. No problems   2. Slight problems   3. Moderate problems   4. Severe problems   5. Extreme problems |
| Assesses HRQoL in patients with cancer   - Scores range from 0 to 100 - Functional scales and GHS/QoL: Higher score = better health or function - Symptom scales: Higher score = worse symptoms | Assesses disease-specific aspects of HRQoL in patients with EC   - Scores range from 0 to 100 - Functional scales: Higher score = better health of function - Symptom scales: Higher score = worse symptoms | Measures global health status across multiple disease states   - - Scale from 0 to 100, in which 100 is the “best imaginable health state” |

EC, endometrial cancer; EORTC QLQ-C30, European Organisation for Research and Treatment of Cancer Quality-of-Life Questionnaire; EORTC QLQ-EN24, EORTC QLQ-Endometrial, 24 questions; EQ-5D-5L, EuroQoL 5 dimensions, 5 levels; GHS/QoL, global health status/quality of life.

*PRO assessment schedule*

HRQoL was assessed on day 1 of each treatment cycle (cycles were 21 days for the LEN+PEMBRO arm and TPC with doxorubicin, and 28 days for TPC with paclitaxel) until the end-of-treatment discontinuation visit. Following the end-of-treatment discontinuation visit, patients were asked to complete the questionnaires during follow-up, for the equivalent of 4 cycle lengths.

The PRO assessment dates were mapped into different time points (baseline to week 114 by every 3 weeks) according to specified actual visit time windows. If there were multiple PRO collections within the same window, the closest collection to the target date was used. If multiple assessments were available on the same day, the most recent was retained.

*Completion and Compliance*

An instrument was considered complete if at least one valid score was available according to the missing item rules outlined in the scoring manual for the instrument. Completion rate was defined as the percentage of number of patients who completed at least one item divided by the number of patients in the HRQoL full analysis set. Compliance rate was defined as the percentage of observed visits divided by the number of eligible patients who were expected to complete the PRO assessment not including patients missing by design (due to death, discontinuation, translation not available, etc).

*Change from Baseline*

To assess changes from baseline, a constrained longitudinal data analysis model was used, with the PRO score as the response variable, and treatment, time, treatment-by-time interaction, and stratification factors used for randomization (MMR status; geographic region, ECOG PS, prior history of pelvic radiation) as covariates. The treatment difference in terms of least square mean change from baseline to the primary timepoint was estimated from this model together with 95% confidence interval.

*Time to Deterioration Analyses*

|  | Time to First Deterioration | Time to Definitive Deterioration |
| --- | --- | --- |
| Definition | - - Time from treatment onset until a worsening of condition     - GHS/QoL, functional scales: decrease in score     - Symptom scales: increase in score | - - Time from treatment onset until a worsening of condition with no subsequent recovery     - GHS/QoL, functional scales: decrease in score     - Symptom scales: increase in score |
| Threshold for event | - EORTC QLQ-C30 and EORTC EN24: 10 points - EQ-5D-5L VAS: 7 points | - EORTC QLQ-C30 and EORTC EN24: 10 points - EQ-5D-5L VAS: 7 points |

EORTC QLQ-C30, European Organisation for Research and Treatment of Cancer Quality-of-Life Questionnaire; EORTC QLQ-EN24, EORTC QLQ-Endometrial, 24 questions; EQ-5D-5L VAS, EuroQoL 5 dimensions 5 levels visual analog scale; GHS/QoL, global health status/quality of life.

The Kaplan-Meier method was used to estimate the deterioration curves and results are presented graphically. A stratified Cox proportional hazards model with treatment as covariate and stratified by MMR status (pMMR vs dMMR), ECOG performance status (0 vs 1), geographic region (region 1 vs region 2), and prior history of pelvic radiation (yes vs no) with Efron's method of tie handling was used to assess the magnitude of the treatment difference (ie, the HR). The HR, its 95% CI and the 2-sided nominal *P* value from the Wald test were reported. If there were zero events in one of the treatment groups, the 2-sided Wald test was replaced with a 2-sided Score test.

The approach for the time to deterioration analyses assumed noninformative censoring. Patients without documented event of interest were censored at the last date of evaluation. Patients without a baseline score were censored at baseline.

**Table S1. HRQoL Change from Baseline to Week 12 in the pMMR population**

|  | **LEN+PEMBRO** | | | **TPC** | | | **Difference in LS means**  **(95% CI)^a^** | ***P*-value** |
| --- | --- | --- | --- | --- | --- | --- | --- | --- |
|  | **Baseline**  **mean (SD)** | **Week 12**  **mean (SD)** | **Change from baseline to week 12**  **LS mean**  **(95% CI)** | **Baseline**  **mean (SD)** | **Week 12**  **mean (SD)** | **Change from baseline to week 12**  **LS mean**  **(95% CI)** |  |  |
| **EORTC**  **QLQ-C30 GHS/QoL** | 66.56  (21.44) | 60.94  (21.35) | -6.80  (-9.43, -4.17) | 66.64 (22.43) | 62.80 (21.67) | -7.96  (-10.86, -5.05) | 1.16  (-2.49, 4.81) | 0.5316 |
| **EORTC**  **QLQ-C30 Physical Functioning** | 79.56  (19.21) | 71.46  (21.64) | -10.42  (-12.65, -8.19) | 76.58  (20.85) | 72.81  (21.13) | -8.68  (-11.13, -6.23) | -1.74  (-4.99, 1.51) | 0.2931 |
| **EORTC**  **QLQ-EN24 Urological Symptoms** | 14.89  (18.09) | 12.37  (18.29) | -2.20  (-4.28, -0.12) | 16.13  (19.79) | 16.56  (19.29) | 0.78  (-1.55, 3.11) | -2.98  (-5.96, 0) | 0.0496 |
| **EQ-5D-5L**  **VAS** | 74.08  (18.33) | 70.23  (18.63) | -5.35  (-7.59, -3.11) | 74.13 (18.61) | 70.90 (19.77) | -7.41  (-9.85, -4.96) | 2.06  (-1.09, 5.20) | 0.1992 |

^a^Based on a constrained longitudinal data analysis model with the patient-reported outcome scores as the response variable with covariates for treatment by study visit interaction, stratification factors mismatch repair status, Eastern Cooperative Oncology Group performance status, geographic region, and prior history of pelvic radiation.

CI, confidence interval; EORTC QLQ-C30, European Organisation for Research and Treatment of Cancer Quality-of-Life Questionnaire; EORTC QLQ-EN24, EORTC QLQ-Endometrial, 24 questions; EQ-5D-5L, EuroQoL 5 dimensions, 5 levels; GHS/QoL, global health status/quality of life; HRQoL, health-related quality of life; LS, least squares; SD, standard deviation; TPC, treatment of physician’s choice; VAS, visual analog scale.

**Figure S1. HRQoL Change From Baseline to Week 12 (A-B) and Over Time (C-F) in the pMMR Population**


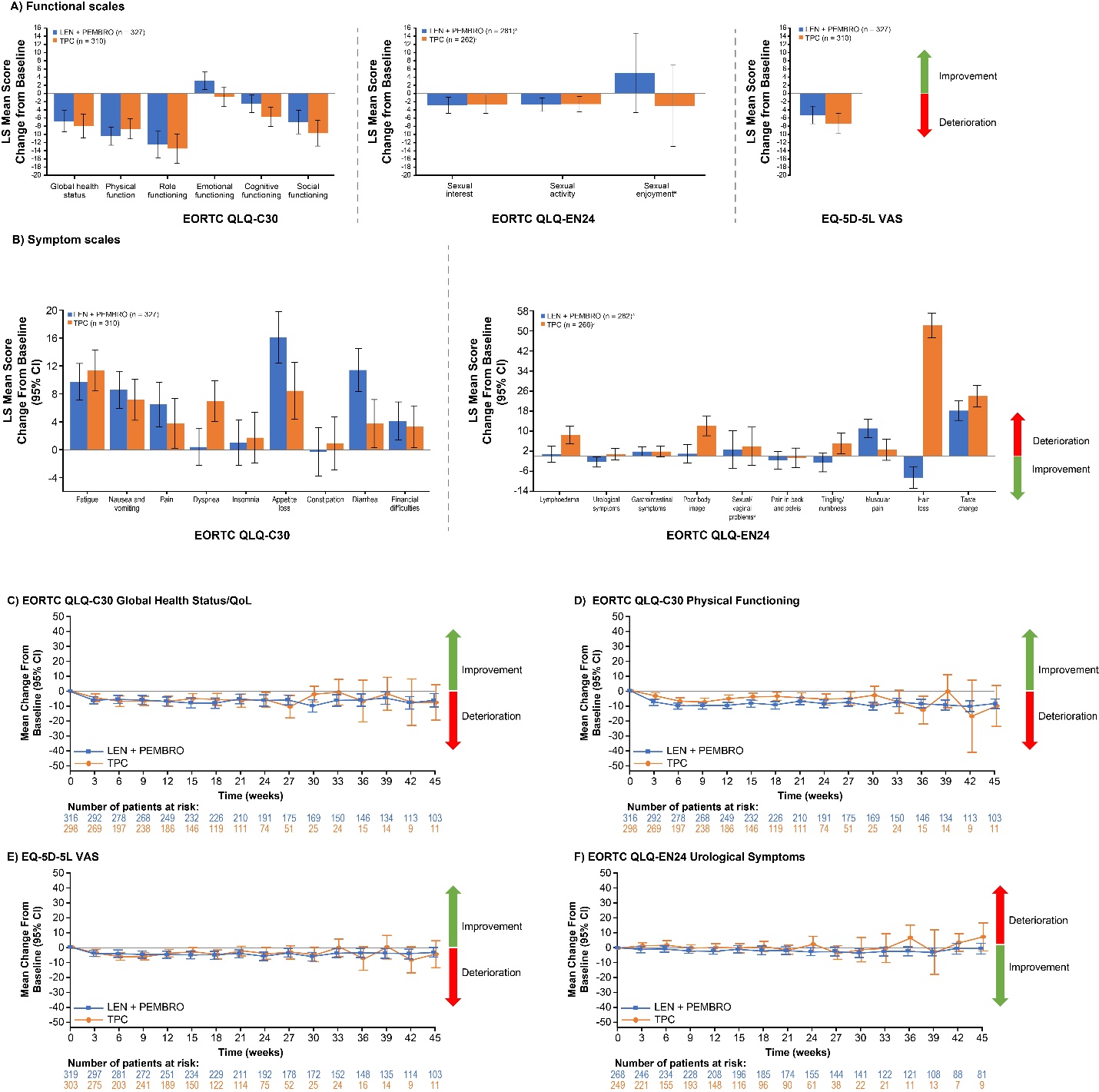


^a^Patient numbers for the sexual enjoyment functional scale and the sexual/vaginal problems symptom scales are LEN+PEMBRO: n = 59, TPC: n = 51.

CI, confidence interval; EORTC QLQ-C30, European Organisation for Research and Treatment of Cancer Quality-of-Life Questionnaire; EORTC QLQ-EN24, EORTC QLQ-Endometrial, 24 questions; EQ-5D-5L, EuroQoL 5 dimensions, 5 levels; GHS/QoL, global health status/quality of life; HRQoL, health-related quality of life; LEN, lenvatinib; PEMBRO, pembrolizumab; pMMR, mismatch repair proficient; TPC, treatment of physician’s choice; VAS, visual analog scale.
